# Associations of moderate alcohol intake with cerebrospinal fluid biomarkers of Alzheimer’s disease: data from the ALBION study

**DOI:** 10.1007/s00394-025-03651-8

**Published:** 2025-04-01

**Authors:** Archontoula Drouka, Klairi-Despoina Ntetsika, Dora Brikou, Eirini Mamalaki, Eva Ntanasi, Stylianos Chatzipanagiotou, Yian Gu, Nikolaos Scarmeas, Mary Yannakoulia

**Affiliations:** 1 Department of Nutrition and Dietetics, Harokopio University, Athens, 17671 Greece; 2 Department of Neurology, Medical School, Aiginition Hospital, National and Kapodistrian University of Athens, Athens, 11528 Greece; 3 Department of Medical Biopathology and Clinical Microbiology, Aiginition Hospital, Athens Medical School, National and Kapodistrian University, Athens, 11528 Greece; 4 The Gertrude H. Sergievsky Center, Department of Neurology, Taub Institute for Research in Alzheimer’s Disease and the Aging Brain, Columbia University, New York, NY 10032 USA

**Keywords:** Alcohol consumption, Mediterranean-alcohol dietary pattern, Alzheimer’s disease, Amyloid beta, Cerebrospinal fluid, Neurodegeneration biomarkers

## Abstract

**Purpose:**

According to a WHO statement, it has been asserted that there is no safe level of alcohol consumption regarding human health. Nevertheless, the relationship between alcohol consumption and Alzheimer’s disease (AD) pathology remains unclear. Therefore, we examined whether the frequency and patterns of alcohol consumption could predict neurodegeneration biomarkers in a cohort of middle-aged adults without dementia.

**Methods:**

A total of 195 participants without dementia were included from the ALBION study. Multivariate logistic regression analyses were conducted using drinking frequency subgroups (abstainers, occasional drinkers, and light-to-moderate drinkers) and Mediterranean-Alcohol Dietary Pattern (MADP) adherence subgroups along with cerebrospinal fluid (CSF) AD biomarkers (Tau, phosphorylated tau (PTau) and amyloid beta (Aβ). In these analyses, the abstinence was used as the reference category.

**Results:**

Of the 195 individuals without dementia, 66% were female, with an average age of 65 ± 9.4 years, and they had 13.8 ± 3.6 years of education. Logistic regression analyses revealed that light-to-moderate drinkers (*n* = 51) were associated with higher Aβ positivity [OR: 2.98 (1.29–6.90)] compared to the abstinence (*n* = 117). Additionally, high adherence to the MADP was significantly associated with higher Aβ, Tau/Aβ_42,_ and PTau/Aβ_42_ ratios positivity compared to the abstinence.

**Conclusion:**

Light-to-moderate alcohol intake was associated with higher Aβ deposition in middle-aged individuals without dementia, compared to abstinence. High adherence to the MADP, which indicates low-to-moderate red wine consumption distributing over the week with meals, was associated with a higher Aβ and Tau/Aβ and PTau/Aβ positivity. Therefore, the management of alcohol consumption may help improve AD outcomes even at the preclinical stage.

## Introduction

In accordance with a statement issued by the World Health Organization (WHO) in January 2023, it has been asserted that there is no safe level of alcohol consumption in terms of human health [1]. A Paneuropean study conducted in 2021 concluded that the consumption of alcohol at a light to moderate level (1–2 drinks per day) was responsible for 23,300 new cases of cancer [2]. However, on the opposite side, it has been argued that the health effects of alcohol are significantly influenced by the quantity consumed, and the drinking patterns. As an example, there is evidence indicating that consuming certain alcoholic beverages in low amounts, along with meals or in specific patterns such as the Mediterranean-Alcohol Dietary Pattern (MADP), may have potential benefits for cardiovascular health [3–5]. In any way, excessive alcohol consumption leads to conditions such as alcoholic liver disease, malnutrition, pancreatitis, brain diseases, and fetal alcohol spectrum disorder in cases of alcohol-use disorder [6].

When it comes to cognitive effects, it is well-established that chronic, heavy alcohol consumption causes alcohol-related brain damage [7] and cognitive deficits [8, 9]. With respect to moderate alcohol consumption, observational studies often report a J-shaped relationship between alcohol consumption and cognitive outcomes, including specific and all-cause dementias; low-to-moderate alcohol consumption is often associated with a lower risk of dementia compared to abstention and heavy drinking [10]. Specifically, meta-analyses of studies involving older adults, both prospective and case-control, indicate that low or even moderate levels of alcohol consumption among older individuals may reduce the risk of cognitive decline and Alzheimer’s disease (AD), revealing a non-linear dose-response relationship between alcohol intake and the risk of AD [11–17].

There are several pathways through which alcohol consumption may affect cognitive function. Alcohol may induce aberrant activation of cell death through various mechanisms including apoptosis, necroptosis, pyroptosis, ferroptosis, and autophagy-dependent cell death. Such pathological changes in neurons, individually or in combination, have been observed in the AD [18]. Pathological hallmarks of AD include primarily the accumulation of two proteins: Amyloid beta (Aβ) peptides and abnormally phosphorylated tau (PTau) proteins [19, 20]. These protein aggregates result in the formation of Aβ plaques and neurofibrillary tangles [21, 22], respectively, and induce neuroinflammation and neurodegeneration over years, leading to a multitude of cognitive and behavioral deficits [23]. Monitoring Aβ levels can serve as an early indication of the onset of AD [24], and the identification of factors that have the potential to influence Aβ, Tau and PTau levels may pave the way for future recommendations in the prevention and treatment of AD [25]. Various environmental factors, such as healthy eating habits [26], adequate sleep [27] and high activity levels [28], may delay the Aβ accumulation.

With respect to alcohol consumption, there are limited and conflicting data regarding its association with the accumulation of Aβ [26]. A cross-sectional study including 806 participants, published in 2021, indicated that moderate alcohol consumption (≥ 1 times/week) may have a detrimental influence on cerebrospinal fluid (CSF) AD biomarkers (Aβ burden, Tau/Aβ, and PTau/Aβ ratios) compared to the light drinking (> 0 times/week and < 1 times/week) [29]. Conversely, previous cross-sectional studies, with smaller sample sizes, indicated that moderate alcohol consumption may have a beneficial influence on Aβ burden in individuals without dementia [30, 31]. Lastly, two cross-sectional and one cohort study reported that there was no significant correlation between alcohol consumption and Aβ and/or Tau and PTau deposition [25, 32, 33]. In summary, the existing observational studies examining the association between CSF AD biomarkers and alcohol consumption are limited and have produced mixed results. In addition, none of these observational studies examined other parameters of alcohol consumption, apart from frequency, such as drinking patterns, including the type of alcohol, whether alcohol consumed with or without meals, the distribution of alcohol consumption over the week or overall alcohol consumption patterns.

Therefore, the aim of this cross-sectional study was to examine whether alcohol consumption is associated with CSF neurodegeneration biomarkers (Aβ burden, Tau/Aβ and PTau/Aβ ratios) in middle-aged adults without dementia. We conducted an analysis of alcohol consumption utilizing two distinct metrics, namely (i) frequency of alcohol consumption and (ii) the MADP.

## Methods

### Study design and procedures

Data utilized in this study were obtained from the Aiginition Longitudinal Biomarker Investigation of Neurodegeneration (ALBION) population-based cohort study. This study is conducted at the Cognitive Disorders Clinic of Aiginition Hospital, affiliated with the National and Kapodistrian University of Athens. Its primary objective is to investigate research inquiries pertaining to the preclinical and prodromal stages of AD. A detailed description of the study protocol has been published previously [34]. In summary, neurologists conducted a comprehensive interview and clinical examination. Vital signs and anthropometric measurements were meticulously recorded. Each participant underwent a thorough assessment encompassing various parameters, including demographics (age, education, sex), medical history, social and environmental factors, clinical indicators, nutritional aspects and neuropsychological determinants. This assessment was conducted through a series of questionnaires. Patients diagnosed with dementia at baseline [35, 36] were specifically excluded, as were individuals with neurological, psychiatric or medical conditions known to carry a high risk of cognitive impairment (including but not limited to Parkinson’s disease, multiple sclerosis, Huntington’s disease, Down syndrome, active alcohol or drug abuse or major psychiatric conditions such as major depressive disorder, schizophrenia and bipolar disorder). In addition, participants with contraindications for lumbar puncture, such as use of anticoagulant medication, were excluded.

The ALBION protocol was approved by the Institutional Review Board of the Aiginition University Hospital of Athens. All participants gave written informed consent to participate in the examinations.

### Selected participants

The study comprised 246 individuals aged 40 years or older, who were either referred by other specialists or self-referred to the cognitive disorders outpatient clinic of a tertiary university hospital. These participants were selected based on criteria such as having a positive family history of dementia, expressing personal concerns regarding their cognitive ability, or demonstrating a sincere dedication to advancing medical science. After excluding subjects without CSF (*n* = 2), dietary data (*n* = 43), with alcohol consumption more than guidelines (*n* = 6) or having dementia (*n* = 2) at baseline, the present cross-sectional analysis includes a total of 195 participants.

### Clinical assessment

A specialist neurologist established a clinical diagnosis following a comprehensive standardized neuropsychological assessment. Only individuals having normal cognitive function (NC) or having mild cognitive impairment (MCI), based on well-established diagnostic criteria [37, 38], were included in this study.

### Measurement of CSF indices

Lumbar puncture took place at the baseline visit. All procedures, as well as the collection, processing, and storage of the CSF, were conducted according to international guidelines [39]. CSF analysis was performed using the Roche Diagnostics Elecsys Platform. Using this analysis, a value below 1,000 pg/mL of Aβ_42_ was agreed upon to define amyloid positivity. Regarding PTau/Aβ_42_ and Tau/Aβ_42_, a value above 0,024 pg/mL and a value above 0,28 pg/mL respectively were considered abnormal.

### Assessment of dietary intake

Dietary intake was assessed through four 24-hour recalls, using the multiple-pass method [40]. Trained registered dietitians conducted interviews with participants, requesting comprehensive reports of all foods and beverages consumed on the preceding day. The initial recall was conducted in person, while the subsequent recalls were administered via telephone calls. The telephone-administered recall demonstrated comparable effectiveness to the face-to-face method [41]. Three of the recalls were conducted on weekdays, while one was conducted on a weekend day, aiming to enhance the accuracy of estimating habitual intake over the entire week. Prior knowledge of the recall day was not provided to the participants, ensuring that they did not alter their dietary habits in anticipation of the interview. The recall data were subsequently analyzed for nutrient content using the dietary analysis software Nutritionist Pro™ (2007, Axxya Systems, Texas, USA).

### Evaluation of alcohol intake

For the assessment of alcohol intake, 1 standard drink was defined as any drink that contained 14 g of pure alcohol (1 glass of wine, 330mL of beer or 30mL of spirits etc.) [42]. According to previous epidemiological studies that examined the association between alcohol consumption and the development of dementia, a clear difference was found in the risk of overall dementia or AD among different alcohol frequency groups, including non-drinkers (reference category), light drinkers, moderate drinkers, and heavy drinkers [30, 43–46]. Based on these studies, we categorized our participants into the following drinking frequency subgroups: according to each drinking status - abstainers (0 standard drinks/week), occasional drinkers (≤ 2 standard drinks/week), and light-to-moderate drinkers (> 2 standard drinks/week). Volunteers with alcohol consumption above the previous European guidelines [47], i.e. consumption above 25 g ethanol per day for males and above 15 g ethanol per day for females were excluded (*n* = 6).

Participants’ diet quality was evaluated through a 10-item composite score. This score is based on the MedDiet Score [48] from which alcohol consumption has been removed, making it a modified version of MedDiet Score (mMedDiet Score). In particular, recall data were grouped into specific food groups, namely full- and low- fat dairy products, non-refined cereals (whole bread, pasta, rice, other grain), fruits, vegetables, potatoes, red meat and products, poultry, fish, legumes, added fats, etc. Based on this food grouping, food groups that are presumed to closely characterize the Mediterranean pattern (i.e. non-refined cereals, fruits, vegetables, legumes, potatoes, fish and olive oil) were assigned scores ranging from 0 to 5, based on the consumption frequency. In specific individuals who reported no consumption were assigned a score of 0, and scores ranging from 1 to 5 are assigned based on frequency of consumption, from rare to daily. For those foods that are presumed to diverge from this diet pattern (i.e. meat and meat products, poultry and full-fat dairy products), participants were assigned scores on a reverse scale. Total mMedDiet Score ranges from 0 to 50, with higher values indicating greater adherence to the Mediterranean diet (MD) pattern.

### Assessment of the mediterranean alcohol-drinking pattern (MADP)

Based on alcohol consumption data, a 0-to-9-points score was calculated to capture the adherence to the MDPA [49]. MDPA adherence is based on the following parameters of alcohol consumption: (a) moderate intake, (b) distribution over the week, (c) preference for wine, (d) preference for red wine, (e) consumption with meals, (f) low consumption of spirits, and (g) avoidance of excess drinking occasions [49]. Specifically, a participant’s score for MADP is positive for moderate alcohol intake, alcohol intake spread out over the week, low spirit consumption, wine preference, red wine consumption, wine consumed during meals and avoidance of binge drinking. MADP score ranged from 0 to 9. Abstainers have a MADP score of 0 and within alcohol consumers, a higher MADP score indicates a more preferred pattern of drinking alcohol. Specifically, 1–4 defined as low-to-moderate MADP adherence and 5–9 defined as high MADP adherence. To the best of our knowledge, the MADP score was also used in two cohort studies (PREDIMED and SUN) [4, 5, 50] which explored the association between alcohol consumption patterns and cardiovascular disease (CVD), including the incidence of CVD, atrial fibrillation and arterial hypertension.

### Statistical analysis

Statistical analyses were performed using IBM SPSS 26 software. Type I probabilistic error was defined as *p* ≤ 0.05. Normally distributed continuous variables are presented as mean values ± standard deviation and comparisons were performed using one-way ANOVA test. In addition, for categorical variables, group differences were examined with chi-squared test.

Multivariate logistic regression analyses with drinking frequency subgroups (including never, occasional drinkers, and light-to-moderate drinkers) and MADP adherence subgroups (abstainers, low-to-moderate adherence, high adherence) as the independent variables and CSF AD biomarkers (normal versus abnormal) as the dependent variables were conducted. In these analyses, the abstainers subgroup was used as the reference category. Regressions were conducted initially without confounders (Model 1) and were subsequently adjusted for years of education, sex and participants’ age (Model 2), as well as for total energy intake and diet quality index (mMedDiet Score) (Model 3). Sex was used as dichotomous variable; education, age, total energy intake and diet quality index were treated as continuous variables.

## Results

### Characteristics of participants

Table 1 shows baseline characteristics for all participants, as well as by drinking frequency categories (abstainers, occasional drinkers, light-to-moderate drinkers). Of the 195 individuals without dementia at baseline (abstainers *n* = 117, occasional drinkers *n* = 27, light-to-moderate drinkers *n* = 51), the average age was 65 ± 9.4 years, they had 13.8 ± 3.6 years of education and the majority of them were female (66%). Light-to-moderate drinkers were more frequently men and had more years of education than abstainers and occasional drinkers. Furthermore, half of the volunteers had pathological Aβ deposition, whereas a smaller proportion of them had pathological ratios of Tau/Aβ_42_ (29%) and PTau/Aβ_42_ (30.1%). CSF biomarkers did not show significant differences across the different alcohol consumption categories. With respect to dietary intake, light-to-moderate drinkers had higher energy intake compared to abstainers and occasional drinkers (2041 ± 544 kcal compared to 1667 ± 474 kcal and 1628 ± 421 kcal, respectively, *p* < 0.0001). Light-to-moderate drinkers had higher adherence to the MADP compared to the occasional drinkers. Lastly, adherence to the mMedDiet Score did not differ across the different alcohol consumption categories.

**Table 1:** Baseline characteristics of all participants and stratified by the frequency of alcohol consumption

Variables	Total Sample (*n* = 195)	Abstainers (*n* = 117)	Occasional drinkers (*n* = 27)	Light-to-moderate drinkers (*n* = 51)
Age (years)	64.7 ± 9.4	64.7 ± 9.1	62.0 ± 8.6	66.0 ± 10.4
Sex (% Female)^a^	66.2%	70.9%	81.5%	47.1%
Education (years)^a^	13.8 ± 3.6	13.3 ± 3.4	13.9 ± 3.4	15.0 ± 3.7
B-Amyloid (% pathological)	50%	45.7%	44.4%	62.7%
Tau/B-Amyloid (% pathological)	29.4%	25.0%	33.3%	37.3%
PTau/B-Amyloid (% pathological)	30.1%	25.9%	34.6%	37.3%
Total energy intake (kcal/day)^a^	1753.6 ± 510.0	1666.8 ± 473.9	1628.1 ± 421.3	2040.5 ± 543.9
MADP (%Low-moderate adherence/ %High adherence) ^a, b^	14.1%/ 25.1%	-	55.6%/ 25%	44.4%/ 75%
mMedDiet Score	28.7 ± 5.7	28.6 ± 5.9	29.6 ± 4.9	28.6 ± 5.8

a) *p* < 0.01

b) Compared adherence to MADP only between participants who consume alcohol

kcal: kilocalories; MADP: Mediterranean Alcohol Drinking Pattern; mMedDiet Score: modified Mediterranean Diet Score; PTau: phosphorylated Tau

Continuous variables are presented as mean values ± standard deviation and categorical variables as relative (%) frequencies

### Alcohol consumption and CSF AD biomarkers

Multivariate logistic regression analyses revealed that the light-to-moderate drinkers (weekly alcohol intake > 2 standard drinks) were significantly associated with higher Aβ positivity compared to the non-drinking group [OR: 2.98 (1.29–6.90), *p* = 0.011], even after controlling for potential confounders (Table 2), while occasional drinking (weekly alcohol intake ≤ 2 standard drinks) was not related to Aβ positivity (Table 2). Furthermore, occasional drinking was associated only with PTau/Aβ_42_ ratio positivity [OR: 2.91 (1.03–8.23), *p* = 0.045] when the model was adjusted for age, sex and education (Model 2, Table 2), and the association was lost when the model was further adjusted for total energy intake and the diet quality index. Drinking frequency subgroups were not associated with Tau/Aβ_42_ positivity.

**Table 2:** Association between alcohol consumption frequency and CSF AD biomarkers

CSF biomarkers		Occasional drinkers		Light-to-moderate drinkers		Abstainers
		OR (95% CI)	p	OR (95% CI)	p	
**Aβ**_**42**_	Model 1	0.97 (0.42–2.26)	0.949	2.05 (1.04–4.03)	**0.038**	Ref.
	Model 2	1.24 (0.51–3.02)	0.630	2.45 (1.18–5.11)	**0.017**	
	Model 3	1.25 (0.48–3.26)	0.643	2.98 (1.29–6.90)	**0.011**	
**Tau/Aβ**_**42**_	Model 1	1.52 (0.61–3.76)	0.367	1.80 (0.89–3.67)	0.104	Ref.
	Model 2	2.65 (0.95–7.36)	0.062	2.04 (0.89–4.71)	0.093	
	Model 3	2.03 (0.67–6.17)	0.213	1.95 (0.77–4.93)	0.160	
**PTau/Aβ**_**42**_	Model 1	1.53 (0.62–3.82)	0.358	1.72 (0.85–3.49)	0.133	Ref.
	Model 2	2.91 (1.03–8.23)	**0.045**	1.92 (0.83–4.43)	0.128	
	Model 3	2.19 (0.70–6.84)	0.176	1.77 (0.70–4.50)	0.230	

Values are odds ratios with confidence intervals between brackets, Aβ_42_, amyloid beta 42; CI, confidence interval; CSF, cerebrospinal fluid; OR, odds ratio; PTau, phosphorylated Tau; ref.: reference category

Model 1: unadjusted model; Model 2: adjusted for age, sex and education in years; Model 3: additionally adjusted for total energy intake and diet quality index

With respect to the Mediterranean alcohol-drinking pattern (MADP), multivariate logistic regression analyses revealed that high adherence to MADP was significantly associated with higher Aβ positivity, as well as higher Tau/Aβ_42_ and PTau/Aβ_42_ ratios positivity compared to the non-drinking group even after controlling for potential confounders (Table 3). Low-to-moderate adherence to MADP was not associated with Aβ burden or CSF biomarkers ratios.

**Table 3:** Association between adherence to the MADP and CSF AD biomarkers

CSF biomarkers		Low-moderate adherence		High adherence		Abstainers
		OR (95% CI)	p	OR (95% CI)	p	
**Aβ**_**42**_	Model 1	1.22 (0.53–2.78)	0.644	2.09 (1.05–4.17)	**0.036**	Ref.
	Model 2	1.52 (0.63–3.67)	0.356	2.48 (1.17–5.22)	**0.017**	
	Model 3	1.51 (0.62–3.66)	0.364	2.30 (1.10–4.81)	**0.027**	
**Tau/Aβ**_**42**_	Model 1	0.83 (0.31–2.25)	0.711	2.47 (1.22–5.01)	**0.012**	Ref.
	Model 2	1.27 (0.41–3.93)	0.677	2.89 (1.28–6.51)	**0.011**	
	Model 3	1.22 (0.40–3.75)	0.731	2.65 (1.18–5.91)	**0.018**	
**PTau/Aβ**_**42**_	Model 1	0.79 (0.29–2.14)	0.643	2.45 (1.21–4.97)	**0.013**	Ref.
	Model 2	1.19 (0.38–3.68)	0.767	2.94 (1.29–6.70)	**0.010**	
	Model 3	1.13 (0.37–3.50)	0.827	2.66 (1.18–5.99)	**0.019**	

Values are odds ratios with confidence intervals between brackets, Aβ_42_, amyloid beta 42; CI, confidence interval; CSF, cerebrospinal fluid; OR, odds ratio; PTau, phosphorylated Tau; ref.: reference category

Model 1: unadjusted model; Model 2: adjusted for age, sex and education in years; Model 3: additionally adjusted for total energy intake and diet quality index

## Discussion

From the several pathways through which alcohol consumption may affect cognitive function, in the present work we examined the potential associations between the main protein hallmarks of AD from CSF samples and alcohol consumption, considering, for the first time, both frequency and drinking patterns, in a study including 195 participants. We found that, compared to alcohol abstinence, light-to-moderate alcohol consumption is associated with Aβ burden. To the best of our knowledge, this is the first time that very low alcohol intake amounts have been examined, even within the low-to-moderate category (maximum daily consumption of 25 g). Additionally, high adherence to the MADP compared to alcohol abstinence, was associated, cross-sectionally, with a higher Aβ burden and Tau/Aβ, PTau/Aβ positivity. Low-to-moderate adherence to the MADP, was not associated with CSF AD biomarkers compared to alcohol abstinence.

Our results were in accordance with a cross-sectional study, that had indicated that moderate alcohol consumption may have a detrimental influence on CSF AD indicators (Aβ burden, Tau/Aβ and PTau/Aβ ratios) compared to the infrequent drinking group [29]. Conversely prior cross-sectional studies indicated that moderate alcohol consumption may have a beneficial influence on Aβ burden [30, 31]. Lastly, two cross-sectional and one cohort study reported that there was no significant correlation between alcohol consumption and Aβ and/or Tau and PTau deposition in individuals without dementia [25, 32, 33]. These inconsistencies in the scientific literature may be explained by the diversity of methodological approaches used to evaluate alcohol consumption (e.g., the differences in measurement and categorization of alcohol consumption). To the best of our knowledge, this is the first study that evaluated alcohol consumption through four 24-h recalls, allowing us to obtain detailed information on the frequency of alcohol consumption and calculate specific patterns.

Additionally, as far as we are aware, none of the previous studies have examined the potential associations between patterns of alcohol consumption and cognitive health. Interestingly, in the field of CVD, prior cohort studies have shown beneficial associations between higher adherence to the MADP and cardiovascular health [4, 5]. However, adherence to the MADP in our study was associated with pathological neurodegeneration biomarkers, possibly due to the influence of alcohol intake on our data. Specifically, since no association was found between wine preference or drinking only with meals (MADP parameters not directly influenced by ethanol amount) and CSF AD biomarkers (data not shown), we presume that the results for the MADP primarily reflect the effect of alcohol intake. Consequently, it would be worthwhile to investigate potential associations between MADP adherence and cognitive function in future studies, which may encompass larger sample sizes or participants with higher levels of alcohol consumption.

The current analysis has notable strengths, including the assessment of dietary intake occurred using 24-h recalls, which allowed us to obtain detailed information on alcohol consumption frequency and patterns, as well as MD adherence and to calculate total energy intake. Moreover, using 4 dietary recalls (3 weekdays and 1 weekend day) minimized the effect of random error (day-to-day variability in dietary intake) and ensured a reflection of usual dietary intake. In addition, this analysis introduces the MADP score for the first time in the field of cognitive health; until now, this score had only been used in the field of CVD. Furthermore, the utilization of automated methods for assessing CSF biomarkers, which appear to align well with amyloid PET scan imaging compared to previously employed assays [51]. Clinical evaluation was carried out by clinicians with subspecialty training and considerable experience in the cognitive disorders field. Additionally, another strength of the study was that participants were self-referred due to memory concerns or a positive family history of late-onset AD dementia. Therefore, participants were evaluated at middle age giving us the opportunity to examine preclinical AD biomarkers years before clinical symptoms appear.

Our study presents several limitations that need to be considered for the interpretation of the results. First of all, this was a cross-sectional analysis, which did not allow us to determine causality between alcohol consumption and CSF AD biomarkers. In addition, while we accounted for demographic variables, total energy intake and healthy eating index as covariates, we cannot rule out the possibility that other significant confounding factors were not included. Furthermore, the moderate sample size restricts the generalizability of our results. In particular, the small sample size of the occasional drinker group (*n* = 27) may not allow for the identification of the association between occasional drinking and CSF AD biomarkers.

## Conclusion

The present study showed significant associations between both drinking frequency and patterns and CSF AD biomarkers in cognitively intact adults, potentially increasing our understanding of how alcohol consumption might influence vulnerability to AD. Specifically, light-to-moderate alcohol consumption was associated with increased Aβ burden. Furthermore, high adherence to the MADP compared to alcohol abstinence, was associated, cross-sectionally, with a higher Aβ burden and Tau/Aβ, PTau/Aβ positivity. Therefore, the management of alcohol consumption may help improve AD outcomes even at the preclinical stage. Our results were in accordance with the WHO statement that there may be no safe level of alcohol consumption in terms of human health.
